# Augmenting the Future Liver Remnant Prior to Major Hepatectomy: A Review of Options on the Menu

**DOI:** 10.1245/s10434-025-17607-z

**Published:** 2025-06-08

**Authors:** Paul Wong, Peter Vien, Jonathan Kessler, Kelly Lafaro, Alice Wei, Laleh G. Melstrom

**Affiliations:** 1 Division of Surgical Oncology, Department of Surgery, City of Hope Medical Center, Duarte, CA USA; 2 Division of Interventional Radiology, Department of Diagnostic Radiology, City of Hope Medical Center, Duarte, CA USA; 3 Division of Hepatobiliary and Pancreatic Surgery, Department of Surgery, Johns Hopkins University, Baltimore, USA; 4 Hepato-Pancreato-Biliary Service, Department of Surgery, Memorial Sloan Kettering Cancer Center, New York, USA

**Keywords:** Future liver remnant, Hepatectomy, Portal vein embolization, Portal vein ligation, Liver venous deprivation, Associating liver partition and portal vein ligation for staged hepatectomy, Radioembolization

## Abstract

The optimization of the future liver remnant (FLR) is paramount in improving outcomes for patients undergoing liver surgery, as post-hepatectomy liver failure remains a major source of postoperative morbidity and mortality. A wide collection of techniques has been introduced with the goal of augmenting the FLR prior to hepatectomy for primary and secondary liver malignancies, and these modalities include portal vein embolization (PVE), portal vein ligation (PVL), liver venous deprivation (LVD), associating liver partition and portal vein ligation for staged hepatectomy (ALPPS), and radioembolization (e.g., Y-90). There are advantages and drawbacks for each of these methods regarding the capacity for FLR hypertrophy sufficient for resection, perioperative morbidity/mortality, and long-term oncologic outcomes. In the context of technical variations when performing the procedures, there have been comparative studies between the various methods of FLR optimization, however, not many in a controlled fashion. Results from ongoing and future randomized controlled trials will help refine these techniques, directly compare outcomes, and personalize strategies based on patient-specific factors. In this review, the benefits of the various FLR augmentation approaches are summarized and the current literature and trials are reviewed.

Over the past several decades, the field of liver surgery has witnessed significant advancements which have resulted in marked improvements in patient safety and outcomes. The adoption of intraoperative techniques, such as maintaining a low central venous pressure and the Pringle maneuver, have aimed to minimize intraoperative bleeding, while postoperative repletion of phosphorus has reduced morbidity.^1–3^ However, the major source of postoperative morbidity and mortality following major hepatectomy remains the development of post-hepatectomy liver failure (PHLF).^4–6^

Historically, the definition of PHLF has had considerable variation that spans across different countries and hospital settings. Several proposed definitions of PHLF have garnered popularity, including the Model for End Stage Liver Disease (MELD) score, “50-50” criteria (bilirubin >50 μL/L and prothrombin time <50% on POD5), and the peak bilirubin criteria (>7 mg/dl) that leverage laboratory values to gauge postoperative hepatic function.^7–9^ The International Study Group of Liver Surgery (ISGLS) proposed a standardized definition and grading in 2011 that described PHLF as a postoperatively acquired decrease in synthetic, excretory, and detoxifying function of the liver represented by increased INR and serum bilirubin on or after POD5.^10^ Notably, when the ISGLS definition was compared to the peak bilirubin and 50-50 criteria, it was the least likely to predict major complications or mortality following hepatectomy.^11^ However, a prospective validation of the ISGLS definition found similar performance to the peak bilirubin and 50-50 criteria, especially when split into Grades B and C to stratify the degree of PHLF.^12^

As measured by the ISGLS definition, the incidence of PHLF has been suggested to be around 8–12%.^13^ Predictors of PHLF include the need for major hepatectomy resulting in inadequate liver remnant, neoadjuvant chemotherapy, and various patient-related factors, such as obesity, diabetes, cirrhosis.^14^ In patients with PHLF requiring changes to clinical management and intervention (Grades B and C), the associated mortality rates are 13% and 54%, respectively. Thus, the need to mitigate the risk of PHLF has spurred the more recent efforts to optimize the future liver remnant (FLR) prior to surgery.^15,16^

The concept of future liver remnant centers on the idea that a safe volume of functional liver with preserved hepatic vascular inflow, outflow, and biliary drainage is needed following liver resection.^17^ This is paramount in all major liver resections, and especially in oncologic resections of primary hepatic malignancies and metastatic liver tumors. In principle, a preoperative calculation of remaining functional liver is conducted after accounting for the volume that is occupied by the tumor. High-quality cross-sectional imaging is needed prior to planned liver resection to identify the anatomical relationships between the hepatic vasculature and the tumor. These preoperative imaging studies are usually conducted with computed tomography (CT) or magnetic resonance imaging (MRI), with noncontrast, arterial, and venous, and delayed phases.

There are several methods of estimating the volume of the FLR. One such method calculates the measured future liver remnant (mFLR) as a proportion of the total functional, nontumoral liver (Fig. 1).^17^ While easy to calculate, this method is agnostic to patient body size and has been shown not to correlate accurately to hepatic metabolic demands, especially in cases of parenchymal atrophy.^18^ The second method, described by Vauthey et al., calculates the total estimated liver volume (TELV) as a function of body surface area (BSA), which is subsequently used to calculate the standardized future liver remnant (sFLR).^19,20^ This series of calculations utilizes patients’ respective height and weight compositions to provide a standardized estimation that can be used to compare sFLRs across various individuals. In contrast to mFLR, sFLR does not incorporate tumor volume and is not confounded by liver disease, biliary dilatation, or tumor number/size.^18,21^ In cases where resectability is precluded by an insufficient FLR, there are several established strategies to optimize the FLR, including portal vein embolization (PVE), liver venous deprivation (LVD), associating liver partition and portal vein ligation for staged hepatectomy (ALPPS), and Yttrium-90 (Y-90) radioembolization (Table 1; Fig. 2).

**Fig. 1:**
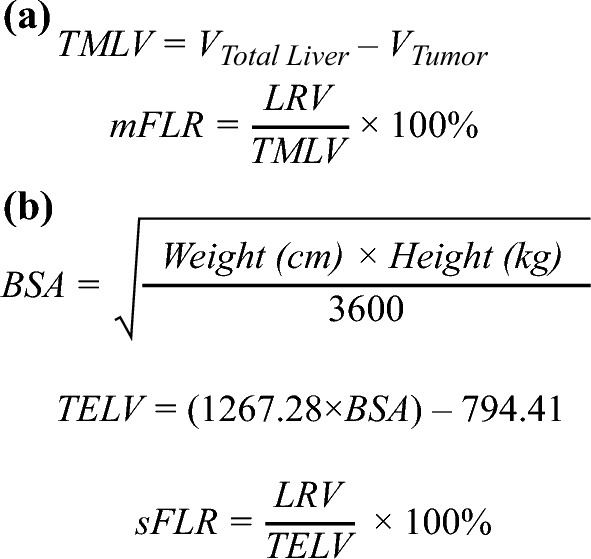
(**a**) Calculation of the future liver remnant volume as a proportion of the total functional, non-tumoral liver and (**b**) standardized total liver volume as a function of body surface area (Vauthey et al.).^19,20^ *BSA* body surface area; *LRV* liver remnant volume; *mFLR* measured future liver remnant; *sFLR* standardized measurement of future liver remnant; *TELV* total estimated liver volume; *TMLV* total measured liver volume; *V*_*Total Liver*_ Volume of entire liver; *V*_*Tumor*_ Volume of tumor^17^

**Table 1:** Strategies to augment the future liver remnant prior to hepatectomy

Procedure	Mechanism of action	Indications/contraindications	Advantages/limitations
Portal vein embolization	Increased portal pressure, local hypoxemia, and inflammation stimulate a regenerative response via release of intrahepatic growth factors and cytokines	Indications: patients without adequate FLR for major hepatectomy, first stage procedure of TSH and ALPPS Contraindications: extensive ipsilateral tumor thrombus, clinically evident portal hypertension	Advantages: relatively low morbidity/mortality; overall morbidity of 2.2%, with 0% procedure-related mortality^32^ Limitations: delay in second surgery procedure since it is performed after ~4–6 weeks, up to 20% of patients fail to undergo planned surgery because of inadequate FLR or tumor progression^38^
Liver venous deprivation	Dual vascular occlusion of portal and hepatic veins creates a more significant reduction in blood flow to the embolized lobe and an increase in pressure that promotes the formation of intrahepatic collaterals, leading to a stronger compensatory contralateral response	Indications: patients without adequate FLR for major hepatectomy Contraindications: extensive ipsilateral tumor thrombus, clinically evident portal hypertension	Advantages: high rate of FLR regeneration Limitations: delay in second surgery procedure since it is performed after ~4–6 weeks, percutaneous approach requires multiple transhepatic accesses in cases of multiple hepatic veins or anatomic variants; hepatic vein anatomic variants present in up to 70% of patients^62^
ALPPS	Ligation of portal vein branch feeding the tumor-bearing liver redirects blood to FLR, leading to compensatory hypertrophy driven by increased blood flow and portal pressure. Parenchymal transection generates a robust regenerative stimulus, likely involving intrahepatic growth factors and cytokines	Indications: patients without adequate FLR for major hepatectomy and need significant hypertrophy; salvage procedure after PVE failure Contraindications: unresectable liver metastases in the FLR, severe portal hypertension, anesthesia risks, extensive comorbidities	Advantages: second surgery performed after only 1–2 weeks and can often be conducted within same hospitalization; effective even after failure of portal vein embolization Limitations: severe complication (Clavien-Dindo ≥IIIb) rate of 27% and 90-day mortality rate of 9%^87^
Y-90 radioembolization	High dose transarterial delivery of Y-90 microspheres induces tumor necrosis that generates a regenerative and inflammatory response. Embolization stimulates hypertrophy through increased portal perfusion and pressure in the nontreated liver segments	Indications: BCLC Stage 0-A with single lesion ≤8 cm,^98^ bridging treatment to transplantation, radiation lobectomy Contraindications: exaggerated hepatopulmonary shunting, pregnancy, propensity for uncorrectable gastrointestinal reflux, recent Capecitabine therapy/breastfeeding^99^	Advantages: minimally invasive, catheter-based delivery; Y-90 microspheres have limited tissue penetration that allows the bulk of the radiation to target tumors instead of normal parenchyma Limitations: slower, more gradual increase in volume compared to PVE

**Fig. 2:**
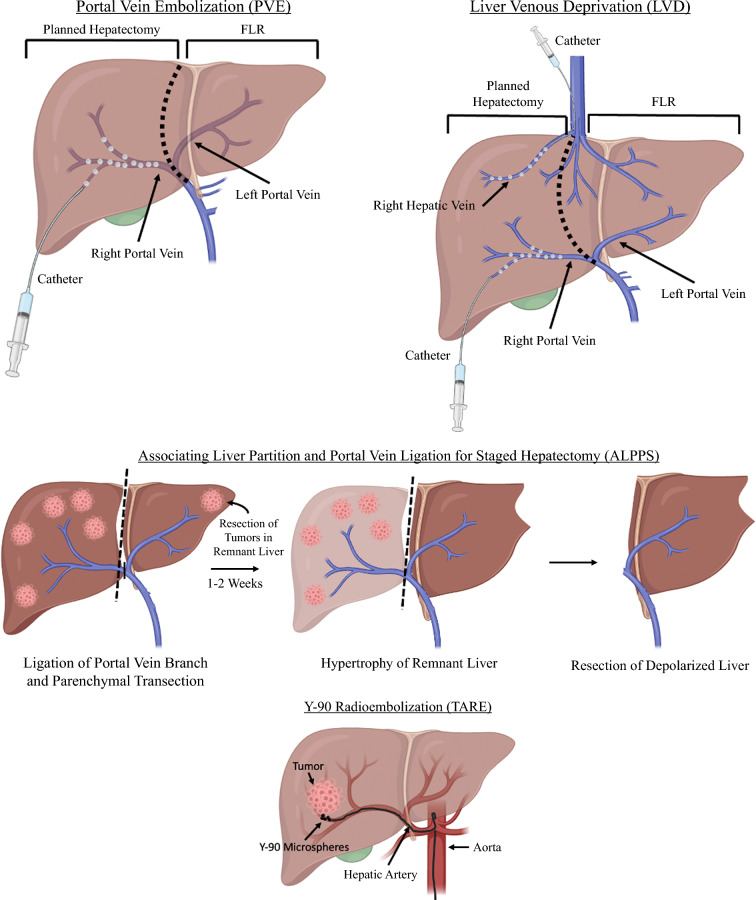
Various techniques to optimize the future liver remnant

The question of whether a patient’s FLR has been optimized prior to liver surgery is addressed through the estimated sFLR. However, the required sFLR to reduce the risk of PHLF is variable among patients and is largely affected by the quality of the native liver parenchyma. For example, a young, healthy patient with normal BMI may be able to tolerate an sFLR of 20%, whereas a patient with chronic hepatitis C or metastatic colorectal liver metastases treated with numerous rounds of neoadjuvant chemotherapy may require an sFLR of 40% or greater to ensure a safe resection. In general, the minimum acceptable sFLR volumes are 20% for normal liver parenchyma, 30% for those treated with neoadjuvant chemotherapy, and 40% for those with cirrhosis.^22–24^ In this review, the benefits of the various FLR augmentation approaches are summarized and the data supporting and comparing strategies are reviewed.

## Portal Vein Embolization

Portal vein embolization (PVE) is a widespread technique for generating contralateral hypertrophy of the FLR in patients undergoing major hepatectomy that was first described by Makuuchi et al.^25,26^ This method of FLR optimization leverages the physiological response that ensues when embolic material is administered into select intrahepatic portal veins to decrease portal vascular flow towards the tumor-involved liver segments and redirected to the anticipated FLR. This effectively leads to atrophy of the ipsilateral liver segments, which will be resected while inducing compensatory hypertrophy of the contralateral liver segments, ultimately increasing the FLR. While the exact mechanism of liver regeneration remains to be elucidated, it is hypothesized that the hypertrophy is associated with the periportal inflammation induced as portal venous flow is diverted from PVE, leading to a regenerative response mediated by the release of intrahepatic growth factors and cytokines.^27^

Furthermore, for patients that undergo PVE, the degree of FLR hypertrophy and kinetic growth rate (KGR) after PVE are additional parameters that can be examined to determine if a patient’s FLR has been optimized.^28^ Kinetic growth rate is defined as the degree of FLR hypertrophy at the initial volume assessment divided by the number of weeks since PVE.^28^ A study by Shindoh et al. demonstrated that a KGR ≥ 2% per week was associated with no cases of PHLF or liver-related 90-day mortality. The study posits that KGR is a better predictor of postoperative morbidity and mortality than sFLR and degree of hypertrophy for PVE.^28^ In addition to increasing the FLR, an important discussion should be had regarding the utility of these strategies as "stress tests" to identify patients capable of tolerating major hepatic resection. This raises the question of whether the absolute increase in FLR should truly be considered as the critical factor. For instance, an sFLR that increases from 18% to 23% may indicate superior regenerative capacity and liver function compared with one that increases marginally from 30% to 31%.

In terms of technique, PVE can be performed either through surgical transileocolic or percutaneous transhepatic or transplenic approach.^29–31^ A meta-analysis by Abulkhir et al. investigated whether transileocolic or transhepatic access approaches yielded differences in hypertrophy response and surgical outcome.^32^ The authors concluded that the increase in remnant liver volume was greater with percutaneous transhepatic approach compared with transileocolic approach (11.9% vs. 9.7%), but a greater proportion of patients underwent surgical resection after transileocolic approach (97% vs. 88%). Furthermore, there was no significant difference seen in rates of major complications post-PVE.

The armamentarium of embolic agents includes gelatin foam, coils, polyvinyl alcohol (PVA) particles, absolute alcohol, and N-butyl-cyanoacrylate (NBCA) glue.^33^ Recent data may suggest that certain embolics may produce superior PVE results. The BestFLR Trial was a randomized controlled trial that compared the liver generation capacity following PVE using different embolic material, namely NBCA with iodized oil versus PVA particles with coils.^34^ Findings from the trial concluded that PVE using NBCA with iodized oil created greater and faster liver hypertrophy than with PVA particles and coils (57% vs. 37% at 28 days). In addition, 87% of patients who received intervention with NBCA and iodized oil had sufficient liver hypertrophy for resection at 2 weeks post-PVE compared with 53% in the PVA particles with coils group.

In general, PVE is a relatively safe procedure that yields few adverse effects with most large studies citing a nearly 0% procedure-related mortality.^32,35^ The major complication rate after the procedure is less than 1%, with complications including infection, venous thrombosis, portal hypertension, biloma, and nontarget embolization.^36,37^ However, there are concerns of delays in the second procedure, because it is performed ~4 to 6 weeks post-PVE, with up to 20% of patients failing to undergo planned surgery because of inadequate FLR or tumor progression.^38^

Given the physiologic rationale of PVE, other potential concerns following the procedure are the growth of the tumor-bearing tissue or increased risk of recurrence following resection. Several studies have highlighted greater tumor growth rate, as measured through Ki-67 proliferation index and mitotic rate, in PVE patients.^39,40^ Margonis et al. investigated the implications of KGR following PVE and found an association between KGR ≥1% in the late regeneration phase (8–10 months postresection) and an increased risk of intrahepatic recurrence.^41^ However, a prospective cohort study of 128 patients from Collin et al. found that PVE was not an independent predictor of overall or disease-free survival.^42^ In fact, preoperative lesion count was the only significant predictor of overall mortality and recurrence in their adjusted analyses.

The risk factors associated with PVE efficacy have been described. Notably, Mise et al. constructed a nomogram predicting hypertrophy after right PVE, concluding that higher BMI, two-stage hepatectomy, and prior hepatectomy were independent predictors of lower degree of hypertrophy of segments 2 and 3.^43^ Furthermore, a systematic review found that lower pre-PVE FLR volume, the additional embolization of segment 4, and use of N-butyl cyanoacrylate were predictive of greater degree of hypertrophy following PVE, whereas neither gender nor neoadjuvant chemotherapy demonstrated a difference.^44^

Portal vein ligation (PVL) relies on the same principle as PVE; however, this must be performed during a surgical procedure and may incur additional morbidity. The ligation of the right or left portal vein can be performed via the open or minimally invasive (laparoscopic/robotic) approach. Most would say this is a reasonable approach in the context of not having interventional radiology expertise at the center or during the first stage of a two-stage hepatectomy (TSH) where the left liver is cleared of disease and the right portal vein ligated. One argument against PVL is often the manipulation of the porta hepatis that may make a repeat surgery more challenging due to inflammation and scar in the porta hepatis.

In terms of its ability to induce hypertrophy, Broering et al. compared the efficacy of right PVL and PVE and found the increase of liver volume was significantly greater with PVE compared with PVL (188 ± 81 mL vs. 123 ± 58 mL, *p* = 0.012).^45^ Hospital length of stay was also shorter for patients who underwent PVE (4 vs. 8 days, *p* < 0.01). However, a systematic review and meta-analysis by Isfordink et al. pooled 1953 PVE and 123 PVL patients from 21 studies, stating that there was no difference in the rate of FLR hypertrophy (PVE 43.2%, PVL 38.5%, *p* = 0.39) or post-procedural morbidity/mortality.^46^ In practice, PVE continues to be performed more often than PVL as it is considered to be less invasive and associated with greater hypertrophy.

Several techniques have been described to further increase hypertrophy from PVE. The addition of segment IV embolization to the standard right PVE has been employed to optimize the hypertrophy of segments II/III in anticipation for extended right hepatectomy.^47^ Various reports have shown greater FLR hypertrophy when embolizing segment IV compared to standard right PVE,^47,48^ whereas others describe similar post-PVE increases between the two.^49–51^ Although less popular, the utility of sequential transcatheter arterial chemoembolization (TACE) + PVE has also been assessed. While TACE+PVE is not widely utilized, there are several reports that have demonstrated its feasibility and comparative ability to generate FLR hypertrophy.^52–55^ Compared with patients that underwent PVE alone, Ogata et al. demonstrated a superior increase in FLR volume, rate of hypertrophy, and disease-free survival in TACE+PVE patients, whereas maintaining similar morbidity and mortality.^53^

## Liver Venous Deprivation

Liver venous deprivation (LVD) is a newer percutaneous technique that augments the future liver remnant through simultaneous or staged embolization of the portal and hepatic veins. The rationale for this approach stemmed from observations of insufficient FLR generation after PVE, leading to reports of sequential hepatic vein embolization (HVE) at the ipsilateral hepatic vein.^56–58^ In their initial case series, Hwang et al. were the first to describe the regenerative effects of ipsilateral HVE after PVE. An increase in FLR was seen following sequential HVE that was attributed to greater amounts of liver damage with the combination of PVE + HVE compared with PVE alone.^56^ Even with the successes seen with sequential HVE following PVE, LVD was introduced as a simultaneous embolization of the hepatic vein and ipsilateral portal vein to dismiss the need to wait between sequential embolization procedures.^59^

Various pathophysiological reasons are responsible for the relatively greater FLR generation in the combined technique compared with PVE alone, which include the increase in hepatic damage from the dual embolization and the formation of venous collaterals. With dual vascular occlusion, LVD creates a more significant reduction in blood flow to the embolized lobe, leading to a stronger contralateral compensatory response. In addition, embolization of both the portal and ipsilateral hepatic vein generates an increase in pressure that promotes the formation of intrahepatic collaterals. This development of venous collaterals decreases the hepatic congestion created from outflow impairment that otherwise would limit hepatic regeneration.^60,61^

The first description of the LVD procedure by Guiu et al. featured a percutaneous approach in which the right hepatic vein was embolized using a plug located 1 cm before the junction with the inferior vena cava, and distal branches and collaterals were subsequently occluded with glue.^59^ Despite the novelty of the percutaneous approach, there were several limitations. Importantly, there is a risk of migration of the injected glue through hepatic venous collaterals after initial plug placement.^59,62^ In addition, the percutaneous approach requires multiple transhepatic accesses in cases of multiple hepatic veins or anatomic variants, which can occur in up to 70% of patients. The inability to occlude anatomic variants and collaterals may lead to suboptimal FLR hypertrophy.^62^ The transjugular LVD approach was developed to overcome the limitations in the percutaneous approach by allowing for embolization of multiple hepatic veins and variants with a single access point. In the transjugular technique, the right internal jugular vein is accessed, and a sheath is advanced to the distal right hepatic vein to facilitate the catheterization of the hepatic veins and variants.^62^ Vascular plugs are then inserted into the catheterized hepatic veins/variants and are reinforced with overlapping plugs to optimize embolization and stability. This technique also allows for more distal placement of plugs to prevent central migration, which can lead to challenges with hepatic vein division at the time of surgery.

Several studies have assessed the potential benefits in FLR size and function of LVD compared to PVE, citing greater hypertrophy, kinetic growth rate, and resectability for LVD patients.^63–65^ Importantly, FLR hypertrophy following LVD is accompanied by a rapid increase in FLR function. Using 99mTc-mebrofenin hepatobiliary scintigraphy, Guiu et al. found a 64.3% increase (range 28.1–107.5%) in FLR function at day 21 post-LVD, with the maximum function seen on day 7.^66^ In a comparison between LVD and PVE, there were greater increases in FLR function in LVD patients at days 7, 14, and 21 (54.3% vs. 23.1%, 56.1% vs. 17.6%, 63.9% vs. 29.8%, respectively).^67^ This is further supported by a systematic review and meta-analysis of nine comparative studies that encompassed 557 patients (207 LVD, 350 PVE) which showed that LVD was associated with greater FLR volume following embolization, lower failure of resection rates, and faster kinetic growth.^68^ Furthermore, there were no differences seen in embolization-related complications, overall morbidity, PHLF, or 3-year overall survival. While the LVD technique remains relatively new, there are several ongoing randomized controlled trials which will provide Level 1 evidence in this comparison between LVD and PVE, perhaps shaping the standard of care in the future (Table 2).

**Table 2:** Published and ongoing randomized controlled studies comparing strategies to achieve future liver remnant hypertrophy

Trial name	Disease type	Comparison groups	Outcomes/endpoints	Postembolization results	Postoperative findings	Oncologic outcomes
Sandström et al., 2018 (LIGRO trial)^82, 110^	CRLM	ALPPS (*n* = 48) vs. TSH (*n* = 49)	Primary: resection rate Secondary: complications, R0 resection margin rate, 90-day mortality	Greater FLR volume increase in ALPPS group (68% ± 38 vs. 36% ± 18, *p* < 0.0001) Greater KGR in days 0–7 in ALPPS patients (14.1 ± 6.0 vs. 6.1 ± 5.4, *p* < 0.0001) Greater % of ALPPS patients reached standardized FLR of 30% after 28 days (92% vs. 47%, *p* < 0.0001) Greater resection rate with ALPPS compared to TSH (92% vs. 57%, *p* < 0.0001)	No differences in: (ALPPS vs. TSH) Clavien-Dindo ≥3a complications (43% vs. 43%, *p* = 0.99) 90-day mortality (8.3% vs. 6.1%, *p* = 0.68) R0 resections (77% vs. 57%, *p* = 0.11)	Improved overall survival seen in ALPPS group (median OS, 46 vs. 26 months, *p* = 0.028) Greater % of ALPPS patients found to be tumor-free in the liver at first postoperative follow up (77% vs. 57%, *p* = 0.028)
Jiao et al., 2019 (REBIRTH trial)^115^	CRLM, ICC, HCC, Others (duodenal adenocarcinoma, PNET, germ cell ovarian tumor, endoemtrial carcinoma, breast cancer, leiomyosarcoma)	RALPPS (*n* = 29) vs. PVE (*n* = 28)	Primary: increase in FLR volume Secondary: length of time to volume gain, morbidity, operation length, postoperative liver function	Greater mean % increase in FLR volume for RALPPS group (80.7 ± 13.7% after a median 20 days vs. 18.4 ± 9.8% after 35 days, *p* < 0.001) Greater resection rate with RALPPS compared with PVE (92.3% vs. 66.6%, *p* = 0.007)	No differences in: (RALPPS vs. PVE) Post-PVE/RALPPS complications (23.0% vs. 20.1%, *p* = 0.2) 90-day mortality (3.8% vs. 0%, *p* = 0.99) R0 resections (75% vs. 68.7%, *p* = 0.87)	N/A
Li et al., 2022^116^	Hepatitis B Associated HCC	ALPPS (*n* = 38) vs. TACE + PVE (*n* = 38)	Primary: resection rate, 3-year overall survival Secondary: rates of increase in FLR volume, time to reach to predefined FLR volume for stage-2 hepatectomy, intraoperative data, postoperative mortality and morbidity rates	Greater resection rate in ALPPS group (97.4% vs. 65.8%, *p* < 0.001) Greater daily increase in FLR volume (15.4 mL/day vs. 3.8 mL/day, *p* < 0.001) No difference in final FLR volume or changes in tumor volume following treatment between the two groups	Higher rate of Clavien-Dindo ≥3a complications in the ALPPS group (54.1% vs. 20.0%, *p* = 0.007) No differences in 90-day mortality (5.3% vs. 5.3%) or resection margin (1 cm vs. 1 cm), both *p* > 0.05	Greater 3-year OS rate in the overall ALPPS group (65.8% vs. 42.1%, *p* = 0.036) In patients that underwent surgical resection, no difference seen in OS between ALPPS and TACE + PVE groups (HR 0.8, 95% CI 0.35–1.83, *p* = 0.595)
DRAGON 2 trial (NCT05428735)^117^	CRLM	Combined PVE/HVE vs. PVE alone (accrual goal of 348 patients, *n* = 174 per arm)	Primary: 5-year overall survival, FLR sufficient for resection in week 3 (based on liver volume) Secondary: FLR volume/function, complications, time to surgery, perioperative variables, quality of life, economical evaluation	Pending results	Pending results	Pending results
HYPER-LIV01 trial (NCT03841305)^118^	CRLM	Simultaneous PVE/HVE vs. PVE alone (accrual goal of 64 patients, *n* = 32 per arm)	Primary: percentage of change in FLR volume at 3 weeks after LVD or PVE using MRI or CT scan Secondary: treatment tolerance, postoperative morbidity/mortality, PHLF, resection rate, perioperative complications, blood loss, R0 resection rate, postoperative liver volume, and overall survival	Pending results	Pending results	Pending results
DRAGON-PLC trial (NCT06914648)^119^	Primary liver cancers	Combined PVE/HVE vs. PVE alone (accrual goal of 358 patients, *n* = 179 per arm)	Primary: 5-year overall survival, FLR sufficient for resection in week 3 (based on liver volume)	Pending results	Pending results	Pending results
TANGO-LIVER trial (NCT06050200)^120^	Hepatic malignancies and liver metastases	PVE vs. LVD vs. partial ALPPS (accrual goal of 154 patients divided into 12:71:71 ratio: PVE, LVD and ALPPS groups, respectively)	Primary: percentage of patients with successful resection following sufficient increase in FLR with no post-surgical 90-day mortality Secondary: rate and degree of FLR volume/function, postprocedural complication rate, length of hospital stay	Pending results	Pending results	Pending results
CCGLC-004 Trial (NCT05103007)^121^	HCC	DEB-TACE vs. PVL/PVE+DEB-TACE (accrual goal of 200 patients)	Primary: resection rate, ratio of FLR proliferation Secondary: overall survival, progression free survival	Pending results	Pending results	Pending results

## Associating Liver Partition and Portal Vein Ligation for Staged Hepatectomy (ALPPS)

Similar to TSH, the ALPPS procedure was originally described by Schnitzbauer et al. as a novel technique to achieve rapid induction of sufficient parenchymal hypertrophy in the remnant liver.^69^ The initial surgery features a classical first stage hepatectomy in which lesions are identified through intraoperative ultrasound, and tumors located in the future remnant hepatic lobe are resected. In addition, the portal vein branch feeding the part of the liver that will be resected is ligated, which redirects blood flow and increases portal pressure to generate FLR hypertrophy. Lastly, the liver parenchyma is divided along a predetermined plane guided by intraoperative ultrasound. This partition separates the FLR from the portion of the liver that will be resected, and importantly, the liver is not completely transected with major blood vessels and bile ducts preserved to maintain liver function. This parenchymal transection generates a robust regenerative stimulus, likely involving intrahepatic growth factors and cytokines.^70^ CT volumetry is conducted to measure the growth of the FLR, and the second stage is subsequently performed which features the completion hepatectomy of the remaining disease.

There is considerable variation in surgical technique for the ALPPS procedure across centers.^27,71^ These modifications in technique stem from a need to improve outcomes and decrease the relatively high morbidity and mortality associated with the procedure. Examples of these different approaches include the partial ALPPS,^72^ radiofrequency assisted liver partition (RALPPS),^73^ Mini-ALPPS,^74^ anterior approach ALPPS,^75^ hybrid ALPPS,^76^ and tourniquet ALPPS.^77^ In addition to technical variability, differences in surgical indication for ALPPS procedure have contributed to the heterogeneity in outcomes. In a survey of surgeons in the International ALPPS Registry, 84% of respondents do not reserve performing the ALPPS procedure to patients with colorectal liver metastases and over half (54%) would consider performing an ALPPS for a FLR >30% predicted by volumetry.^71^ This apparent incongruency in practice patterns has highlighted the importance of consensus recommendations for ALPPS with an emphasis on patient selection and standardization of surgical technique.

Compared with the 4- to 6-week period traditionally seen with TSH, the interval period between the first and second surgeries in the ALPPS procedure is generally 1 to 2 weeks.^27^ Thus, the various advantages of ALPPS include the ability to perform both stages of the procedure in the same hospitalization given the short interval between surgeries and the 100% completion rate of the second surgery.^27,78^ In contrast, the main downside of TSH is the possibility of disease progression in the interval period between the two stages, with dropout rates that can be as high as 36%.^79^ The superior completion rate for the ALPPS procedure is supported by various studies demonstrating a greater FLR regeneration when compared to TSH.^80–82^ The LIGRO trial compared the two approaches in a randomized controlled fashion and demonstrated a superior resection rate in ALPPS compared with TSH (92% vs. 57%) with similar complication rates and mortality.^82^ Given its greater regenerative ability, ALPPS has been proposed to be a salvage procedure for patients that fail to generate adequate FLR hypertrophy following portal vein occlusion.^83,84^ In a subgroup of 22 patients in the ALPPS registry that previously failed portal vein occlusion, all of them completed ALPPS with a median FLR growth of 88% and without any reports of PHLF and 90-day mortality.^83^

There are several major drawbacks of the ALPPS procedure, specifically pertaining to its high morbidity and mortality. First, there are concerns that FLR hypertrophy is not necessarily accompanied by an equivalent increase in hepatic function. In their case series, Sparrelid et al. demonstrated a median FLR volume increase of 56.7% on day 6 after stage 1, while there was only a 28.7% increase in FLR function.^85^ Similarly, a multicenter study of 60 patients shared this sentiment that volumetry overestimates FLR function, with a median 78% increase in FLR volume and only a 29% increase in liver function as measured by hepatobiliary scintigraphy.^86^ In addition, the initial reports from the International ALPPS Registry cited a severe complication (Clavien-Dindo ≥IIIb) rate of 27% and 90-day mortality rate of 9%.^87^ Independent risk factors for severe complications were found to be blood transfusion, operating time > 300 min for ALPPS stage I, age >60 years, and non-CRLM disease. Furthermore, Wanis et al. explored center-specific variations in morbidity and mortality for early-adopters of the ALPPS procedure.^88^ There was considerable variability in 90-day mortality (range, 4.2–29.1%) and comprehensive complication index (range, 17–49.8) across centers, and an association was seen between higher volume hospitals and lower risk of mortality.

## Radioembolization

Transarterial radioembolization (TARE), also known as selective internal radiation therapy (SIRT), involves the delivery of radioactive microspheres into the liver’s arterial supply to preferentially treat liver tumors. The pathophysiological basis for radioembolization stems from the differential perfusion patterns of hepatic tumors and normal liver parenchyma. The vast majority of the blood supply to hepatic tumors comes from the hepatic arteries, while liver parenchyma derives most of its supply from the portal vein.^89–91^ In addition, these microspheres also have limited tissue penetration that allows the bulk of the radiation to target tumors.^92^ Thus, the intra-arterial delivery of these microspheres leads to the selective treatment of liver tumors without significantly compromising the function of normal liver parenchyma. Furthermore, TARE has been shown to be effective as both a downstaging modality and bridge to surgical resection or transplantation.^93–97^

Yttrium-90 (Y-90) a Beta-emitting particle, is the most common radiation source used in TARE for the treatment of primary and metastatic liver tumors. It may be bound to either resin or glass microspheres and calibrated for specific radiation dose and sphere number. While Y-90 is indicated for treatment of BCLC Stage 0-A patients with solitary HCC lesion ≤8 cm and used as a bridge to resection or transplantation,^96–98^ its contraindications include exaggerated hepatopulmonary shunting, pregnancy, propensity for uncorrectable gastrointestinal reflux, recent Capecitabine therapy, and breastfeeding.^99^ Aside from the antitumoral effect of the radiation, if given with the appropriate radiation dose, TARE has the additional ability to induce contralateral hypertrophy of the nontreated liver.^100^ This dual-ability of Y-90 radioembolization is important in patients that are initially deemed unresectable as significant contralateral hypertrophy and downstaging can lead to the possibility for surgical resection. In a systematic review of seven studies examining 312 patients, the range of contralateral liver hypertrophy was 26–47% in periods of 44 days to 9 months.^101^ Importantly, these studies were retrospective in nature, were not specifically designed to optimize liver hypertrophy, featured a heterogenous patient population with a mixture of pathologies and underlying liver disease, and had considerable variation in treatment duration and dosage.

There are several potential benefits of Y-90 radioembolization over other liver augmentation strategies. First, Y-90 is among the least invasive options for FLR augmentation and is typically performed as an outpatient procedure with minimal morbidity and risk.^102,103^ Additionally, compared with other methods, Y-90 possesses the dual function of generating contralateral hypertrophy while also treating the tumor in the affected lobe, thereby minimizing the risk of further tumor progression that may render patients unresectable.^96^

The downsides of Y-90 radioembolization pertain to its lack of widespread availability across facilities and ability to efficiently cause hypertrophy. There are various studies that have conducted time-dependent analyses on FLR hypertrophy,^101,104,105^ While Y-90 was able to generate hypertrophy that is similar in magnitude to other methods, the findings suggest that the process may take significantly longer that alternative approaches, on the order of several months.^104,105^ Vouche et al. found 1-, 3-, and 6-month FLR hypertrophy of 7%, 24%, and 35%, respectively.^104^ Bekki et al. demonstrated that, compared with PVE, Y-90 radioembolization yielded a greater degree of hypertrophy (63% vs. 36%) and better tumor control, but PVE patients had a higher resectability rate (85% vs. 64%).^106^ However, other reports have found that Y-90 produced less contralateral hypertrophy than PVE.^107,108^ To further optimize FLR hypertrophy, a preliminary case series has detailed the potential utility of sequential Y-90 and portal vein embolization before major hepatectomy.^109^ While studies report risks of TARE to include liver dysfunction and non-target radiation injury to adjacent abdominal organs and lung, these have been essentially eliminated with modern techniques and are thought to be extremely unlikely when performed treatment is restricted to a single lobe, as is typically done when performed to optimize liver hypertrophy.

## Randomized Controlled Trials Comparing Future Liver Remnant Optimization Strategies

### LIGRO Trial

There remains a paucity of randomized controlled trials comparing the effects of the various FLR augmentation strategies prior to hepatectomy. However, several published and ongoing trials have been established (Table 2). The LIGRO Trial is a prospective, multicenter RCT that featured 97 patients with colorectal liver metastases and a standardized FLR of less than 30% and assessed whether performing ALPPS increases resection rates compared with TSH.^82^ The primary outcome was resection rate following the first stage of the procedure and was found to be higher in the ALPPS group (92% vs. 57%, *p* < 0.0001). This superiority in the ALPPS arm was supported by findings of greater FLR volume increase (68% ± 38 vs. 36% ± 18, *p* < 0.0001), KGR in days 0–7 (14.1 ± 6.0 vs. 6.1 ± 5.4, *p* < 0.0001), and percentage of patients who reached standardized FLR of 30% after 28 days (92% vs. 47%, *p* < 0.0001). Importantly, there were no differences seen in Clavien-Dindo ≥3a complications (43% vs. 43%, *p* = 0.99), 90-day mortality (8.3% vs. 6.1%, *p* = 0.68), or R0 resections (77% vs. 57%, *p* = 0.11).

Following the initial report of findings in the LIGRO trial, several subsequent analyses were published regarding the long-term oncologic outcomes and health economic evaluations between ALPPS and TSH. The trial group found a greater overall survival in the ALPPS group (median OS, 46 vs. 26 months, *p* = 0.028) and higher percentage of ALPPS patients found to be tumor-free in the liver at first postoperative follow-up (77% vs. 57%, *p* = 0.028).^110^ In addition, there did not seem to be a greater rate of rapid recurrence in a subset of the ALPPS group compared to the TSH arm, despite reports of early tumor recurrence in the literature.^111–113^ With 2-year follow-up data, an additional health economic evaluation of the LIGRO trial patients was conducted and did not show any differences in mean cost, life years, and quality-adjusted life years (QALYs) between the treatment groups.^114^

### REBIRTH Trial

The REBIRTH Trial compared the efficacy of radiofrequency assisted ALPPS (RALLPS) vs. PVE in 57 patients with primary or secondary hepatic tumors.^115^ Results from the trial found RALLPS to be more effective than PVE, as seen through a greater mean percentage increase in FLR volume (80.7 ± 13.7% after a median 20 days vs. 18.4 ± 9.8% after 35 days, *p* < 0.001) and higher resection rate (92.3% vs. 66.6%, *p* = 0.007). Despite this, there were no differences seen in post-PVE/RALPPS complication rate, 90-day mortality, and percentage of R0 resections.

### ALPPS Versus TACE + PVE

In patients with hepatitis B associated hepatocellular carcinoma, Li et al. performed a single-center, prospective randomized study to compare resection rates and 3-year overall survival in patients that underwent ALPPS or TACE + PVE.^116^ There was a superior resection rate in the ALPPS group (97.4% vs. 65.8%, *p* < 0.001), along with a greater daily increase in FLR volume (15.4 mL/day vs. 3.8 mL/day, *p* < 0.001). ALPPS patients had a higher rate of Clavien-Dindo ≥3a complications (54.1% vs. 20%, *p* = 0.007), but there were no differences in 90-day mortality or resection margin between the treatment groups (both *p* > 0.05). For oncologic outcomes, patients in the ALPPS group as a whole had a greater 3-year OS rate (65.8% vs. 42.1%, *p* = 0.036). However, in the subset of patients that underwent surgical resection, there was no survival difference between the ALPPS and TACE + PVE groups (HR 0.80, 95% CI 0.35–1.83, *p* = 0.595).

### Ongoing Randomized Controlled Trials

There are several ongoing randomized controlled trials evaluating the comparative efficacy of various liver augmentation strategies and are summarized in Table 2. The DRAGON 2 (NCT05428735) and HYPER-LIV01 (NCT03841305) trials were both created to assess the efficacy of combined PVE/HVE versus PVE alone in patients with planned hepatectomy for colorectal liver metastases.^117,118^ Similarly, the DRAGON-PLC trial (NCT06914648) will compare resectability 3 weeks after embolization and overall survival in patients with primary liver cancers following either combined PVE/HVE or PVE alone.^119^ The TANGO-LIVER Trial (NCT06050200) is planning on including three arms in the study—PVE vs. LVD vs. partial ALPPS—with the primary outcome of resection rate with no post-surgical 90-day mortality following sufficient increase in FLR.^120^ Lastly, the Chinese CCGLC-004 Trial (NCT05103007) is evaluating the comparative benefits of drug-eluting bead transarterial chemoembolization (DEB-TACE) versus PVL/PVE + DEB-TACE in HCC patients, specifically looking at resection rate, FLR proliferation, overall survival, and progression-free survival.^121^

## Conclusions

The optimization of the future liver remnant (FLR) is paramount in improving outcomes for patients undergoing liver surgery, as post-hepatectomy liver failure remains a major source of postoperative morbidity and mortality. A wide collection of techniques has been introduced with the goal of augmenting the FLR prior to hepatectomy for primary and secondary liver malignancies, often in patients initially deemed unresectable. There are advantages and drawbacks for each of these methods regarding the capacity for FLR hypertrophy sufficient for resection, perioperative morbidity/mortality, and long-term oncologic outcomes. In the context of technical variations when performing the procedures, there have been comparative studies between the various methods of FLR optimization, however, not many in a controlled fashion. Results from ongoing and future randomized controlled trials will help refine these techniques, directly compare outcomes, and personalize strategies based on patient-specific factors.
